# The evaluation of monocyte lymphocyte ratio as a preoperative predictor in urothelial malignancies: a pooled analysis based on comparative studies

**DOI:** 10.1038/s41598-019-42781-y

**Published:** 2019-04-18

**Authors:** Xuan Zhu, Shui-Qing Wu, Ran Xu, Yin-Huai Wang, Zhao-Hui Zhong, Lei Zhang, Xiao-Kun Zhao

**Affiliations:** Department of Urology, The Second Xiangya Hospital, Central South University, Hunan Province, People’s Republic of China

**Keywords:** Tumour biomarkers, Tumour biomarkers, Prognostic markers, Prognostic markers, Tumour biomarkers

## Abstract

In recent years, several studies have reported monocyte lymphocyte ratio (MLR) to predict prognosis in various tumors. Our study was performed to evaluate the association between preoperative MLR between prognostic variables in urothelial carcinoma patients. Systematic literature search was conducted in PubMed, Embase, Web of science. The correlation between preoperative MLR and overall survival (OS), cancer specific survival (CSS), disease free survival (DFS)/relapse free survival (RFS), progression free survival(PFS) was evaluated in urothelial carcinoma patients. Meanwhile, the association between MLR and clinicopathological characteristics was assessed. Finally, 12 comparative studies comprising a total of 6209 patients were included for pooled analysis. The hazard ratios (HRs), odds ratios (ORs)and 95% confidence intervals (CIs) were further analyzed as effect measures. The pooled results demonstrated that elevated preoperative MLR indicated unfavorable OS (HR = 1.29, 95%CI = 1.18-1.39, I^2^ = 33.6%), DFS/RFS (HR = 1.42, 95%CI = 1.30–1.55, I^2^ = 0.0%) and CSS (HR = 1.41, 95%CI = 1.29–1.52, I^2^ = 0.0%). Moreover, the pooled results also suggested that elevated preoperative MLR was correlated with high tumor stage (OR = 1.22, 95%CI = 1.07–1.37, I^2^ = 0.0%) in urothelial carcinoma patients. No significant association was found between preoperative MLR and PFS in upper urinary tract urothelial carcinoma (UUTUC) patients. Collectively, elevated preoperative MLR predicted poor prognosis in urothelial carcinoma and have the potential to be a feasible and cost-effective prognostic predictor for management of urothelial carcinoma.

## Introduction

Urothelial carcinoma is defined as the malignancy derived from the mucosal surface of urinary system. Urothelial carcinoma of bladder (UCB) was the most common malignancy in urothelial carcinoma, followed by UUTUC accounting for 5–10% of all urothelial malignancies^1,2^. Urothelial carcimona always means a large toll on human health and huge economic burden for patients or health care systems^3^, due to its high recurrence and malignancy. In spite of advanced surgical techniques, growing expertise, emerging new treatments in recent years, the improvement of long-term survival have barely changed and the treatment decision-making for urothelial carcinoma is still oftentimes challenging^1,4–6^. Therefore, it is essential to develop and validate the potential biomarkers to establish the accurate preoperative risk stratifications and predict the prognosis after treatment.

Inflammation can affect immune surveillance and responses to the treatments for tumors, and there has been increasing evidence indicating that systemic inflammatory responses play an important role in the development, progression and metastasis of malignancies in past decades^7–9^. Many inflammation related biomarkers have been evaluated as prognostic indicators in multiple malignancies, such as platelet to lymphocyte ratio (PLR)^10^, c-reactive protein (CRP)^11^, tumor associated macrophages (TAMs)^12^ and so on. Recently, the lymphocyte to monocyte ratio (LMR) has been reported as a predictor in various tumors^13–15^. Since 2014, emerging studies have reported the preoperative MLR as a potential predictor in UCB or UUTUC patients^16–18^, and Yoshida’s study reported preoperative MLR could be a better predictor in UCB patients, comparing with NLR^19^. However, the role of preoperative MLR is still in controversy for urothelial malignancies, and needs to be validated due to the inevitable discrepancy among the studies. Therefore, we performed this pooled analysis to assess the potential impact of preoperative MLR in patients with urothelial malignancies. Moreover, relationships between preoperative MLR and the clinicopathological characteristics was also assessed.

## Results

### Literature selection

**Figure 1:**
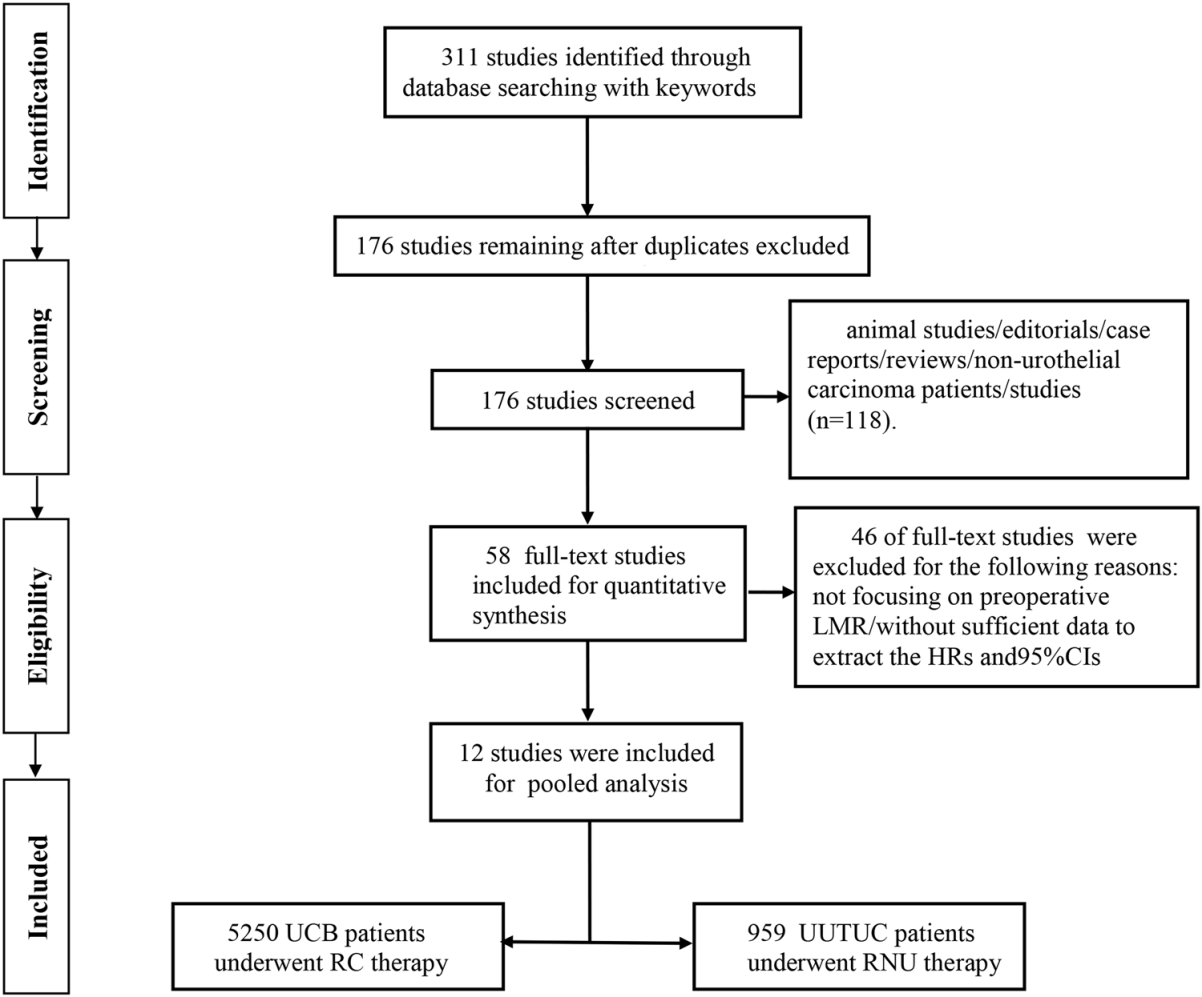
Flow diagram.

There databases (Pubmed, Embase and Web of science) were systematically searched, and 311 studies were initially identified. 176 studies remained after removing 135 duplicates. Subsequent to the screening of titles and abstracts of the remaining 176 studies, 118 studies were excluded for the following reasons: animal studies, editorials, case reports, reviews, non-urothelial carcinoma patients’ studies. Therefore, 58 full-text studies were evaluated for eligibility, and 46 studies were further excluded for not focusing on preoperative MLR or without sufficient data to extract the HRs and 95%CI for prognostic variables. Finally, 12 studies comprising a total of 6209 patients were included in this pooled analysis^16–28^. The flow diagram for literature selection was shown in Fig. 1.

### Characteristics of the included publications

The MLR data were collected before surgery in the included studies. The number of patients ranged from 68 to 4198, with 959 UUTUC patients and 5250 UCB patients included in this pooled analysis. All the patients underwent radical surgery, radical cystectomy was chosen for UCB patients and UUTUC patients were performed with radical nephroureterectomy. MLR was defined as absolute monocyte counts divided by lymphocyte counts, and the cut-off values for MLR ranged from 0.25 to 0.50, while Bhindi’s study reported per 1-log unit as the cut-off value. As for Temraz’s study, there were two kinds of cut-off values to evaluate the potential role of MLR in DFS or OS, respectively^16^. The studies were designed retrospectively and published between 2014 and 2018. With regard to prognostic outcomes assessed, OS was investigated as prognostic endpoint in 10 studies, DFS/RFS in 5 studies and CSS in 5 studies, and PFS in 3 studies. Regarding the quality of included studies, the mean NOS score was 7.3. The detailed characteristics were summarized in Table 1.

**Table 1:** Charateristics of the included studies.

First author(year)	Design	geographical region	Cases number	diagnosis	therapy	Cut-off value	Outcome	Analysis methods	NOS score
Temraz S, *et al*.(2014)	Retrospective	Asian	68	UCB	RC	0.36^a^,0.35^b^	DFS,OS	K	6
Hutterer GC, *et al*.(2015)	Retrospective	Australasian	182	UUTUC	RNU	0.50	OS	M	8
Zhang GB, *et al*.(2015)	Retrospective	Asian	124	UCB	RC	0.25	OS	U	7
Yoshida T, *et al*.(2015)	Retrospective	Asian	181	UCB	RC	0.28	OS	M	8
Bhindi B, *et al*.(2016)	Retrospective	North American	418	UCB	RC	per 1-log unit^c^	RFS,CSS,OS	U	7
Song X, *et al*.(2016)	Retrospective	Asian	140	UUTUC	RNU	0.28	DFS,PFS	M	7
D’Andrea D, *et al*.(2017)	Retrospective	European	4198	UCB	RC	0.29	RFS,CSS,OS	M	8
Altan M, *et al*.(2017)	Retrospective	Asian	113	UUTUC	RNU	0.34	DFS,PFS	M	6
Miyake M, *et al*.(2017)	Retrospective	Asian	117	UCB	RC	0.3	OS,DSS	U	7
Rajwa P, *et al*.(2018)	Retrospective	European	144	UCB	RC	0.41	OS,CSS	M	8
Jan HC, *et al*.(2018)	Retrospective	Asian	424	UUTUC	RNU	0.4	OS,CSS,PFS	M	8
Zhang XK, *et al*.(2018)	Retrospective	Asian	100	UUTUC	RNU	0.33	OS	M	8

UUTUC: upper urinary tract urothelial carcinoma; UCB: urothelial carcinoma of bladder; OS: overall survival; DFS: disease free survival; RFS: recurrence free survival; CSS: cancer specific survival; PFS: progression free survival; RNU: radical nephroureterectomy; RC: radical cystectomy; M: multivariate analysis; U:univariate analysis; K: Kaplan-meier curve; a: cut-off value for OS; b: cut-off value for DFS; c: Log-transformed.

### Prognostic significance of MLR in OS

**Figure 2:**
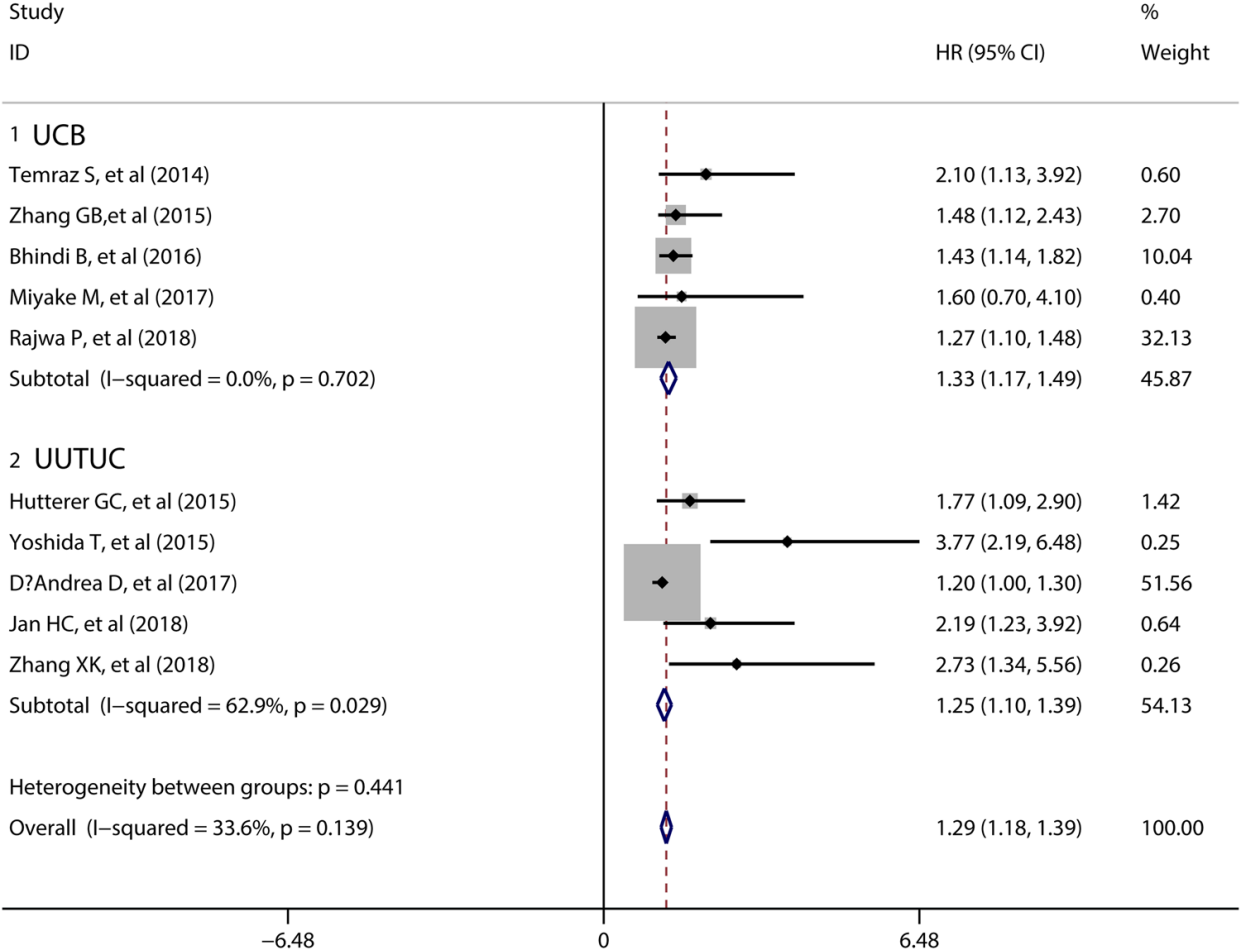
Forest plot evaluating the association between MLR and OS.

10 studies comprising 5956 patients reported the data of MLR and OS in urothelial carcinoma patients. Overall, the pooled results indicated elevated preoperative MLR was correlated with reduced OS (HR = 1.29, 95%CI = 1.18–1.39), without significant heterogeneity among the included studies (I^2^ = 33.6%, p = 0.139) (Fig. 2), Subgroup analyses regarding UCB and UUTUC types were conducted to detect the potential heterogeneity. The pooled results suggested elevated preoperative MLR predicted reduced OS in both UCB patients (HR = 1.33, 95%CI = 1.17–1.49) and UUTUC patients (HR = 1.25, 95%CI = 1.10–1.39). The details were illustrated in Fig. 2.

### Prognostic role of MLR in DFS/RFS

**Figure 3:**
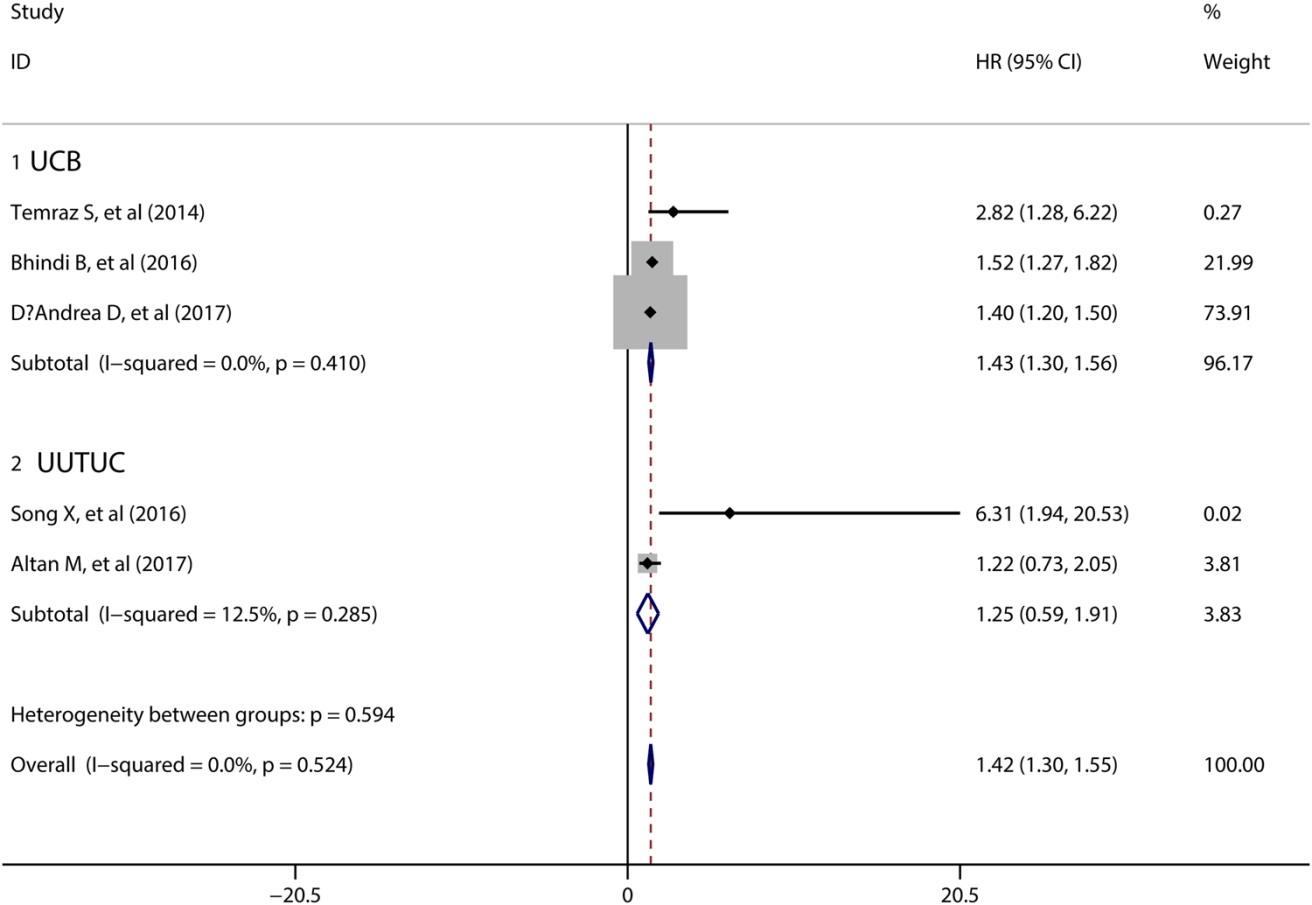
Forest plot evaluating the association between MLR and DFS/RFS.

5 studies involved with 4937 patients assessed the potential role of preoperative MLR in DFS/RFS. The pooled results demonstrated that elevated preoperative MLR predicted poor DFS/RFS in urothelial maligancies (HR = 1.42, 95%CI = 1.30–1.55), without significant between-study heterogeneity studies (I^2^ = 0.0%, p = 0.417) (Fig. 3). Subgroup analysis indicated elevated preoperative MLR was significantly associated with poor DFS/RFS in UCB patients, and no significant association was found between MLR and DFS/RFS in UUTUC patients.

### Prognostic role of MLR in CSS

**Figure 4:**
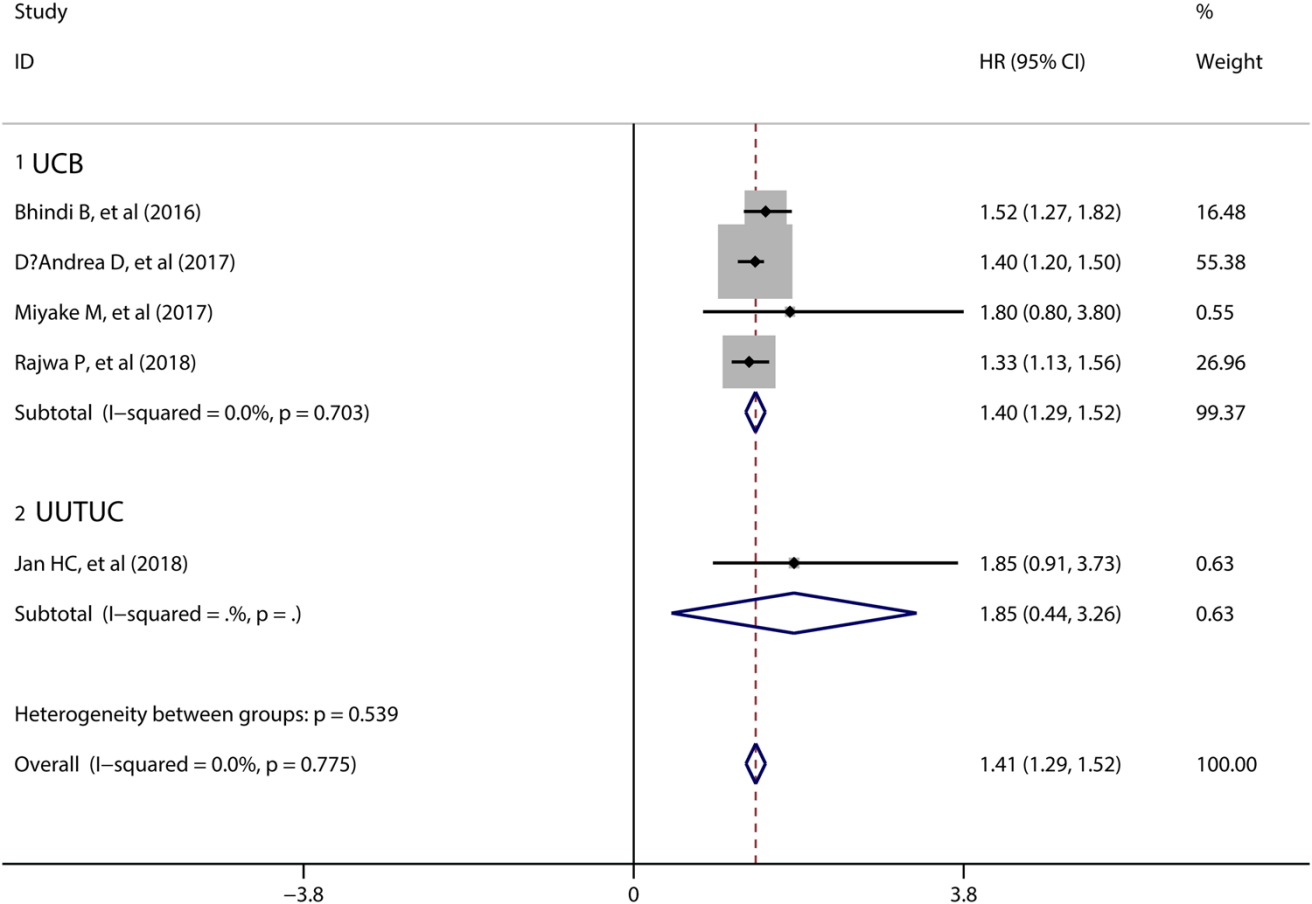
Forest plot evaluating the correlation between MLR and CSS.

5 studies including 5301 patients presented the data about MLR and CSS, and 4 of 5 studies focused the relationship between MLR and CSS in UCB patients. The pooled results showed that elevated preoperative MLR was significantly associated with poor CSS in urothelial patients (HR = 1.41, 95%CI = 1.29–1.52), without significant heterogeneity among the 5 studies (I^2^ = 0.0%, p = 0.775). Only 1 of 5 studies reported the prognostic role of MLR in CSS in UUTUC patients (HR = 1.85, 95%CI = 0.91–3.73) (Fig. 4).

### Prognostic role of MLR in PFS

**Figure 5:**
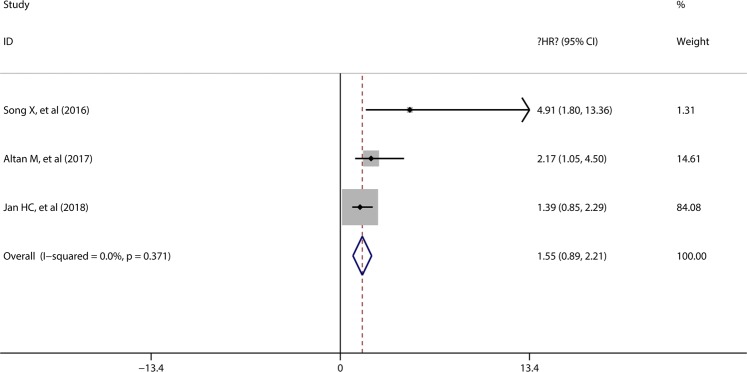
Forest plot evaluating the correlation between MLR and PFS.

3 studies including 677 patients presented the data about MLR and CSS, and all the patients were diagnosed as UUTUC patients. The pooled results showed that no significant association was found between preoperative MLR and PFS in UUTUC patients (HR = 1.55, 95%CI = 0.89–2.21), and significant heterogeneity was not found among the included studies (I^2^ = 0, p = 0.371) (Fig. 5).

### Pooled analysis of MLR and clinicopathological characteristics

Raw data were extracted and calculated for the ORs combined with 95%CIs to assess the impact of preoperative MLR on clinicopathological characteristics in urothelial carcinoma patients (high MLR vs. low MLR). Our pooled results based on pooled analysis suggested elevated preoperative MLR predicted high Tumor stage (≥T2 vs. <T2) in UCB or UUTUC patients (OR = 1.22, 95%CI = 1.07–1.37), without significant heterogeneity (I^2^ = 0, p = 0.661). No significant correlation was found between preoperative MLR and Diabetes, tumor necrosis, multifocality, tumor grade and lymphovascular invasion (LVI). The detailed results were shown in Table 2.

**Table 2:** The association between MLR and clinicopathological characteristics. MLR: high vs. low; Cis: carcinoma *in situ*; LVI: lymphovascular invasion.

Patient charateristics	Number of studies	Number of patients	Effect models	OR (95%CI)	Heterogeneity		
					Chi^2^	I^2^	P
Gender (male vs. female)	6	5168	fixed	0.87 (0.75–0.99)	2.59	0	0.763
Diabetes (yes vs. no)	2	264	fixed	0.66 (0.16–1.17)	0.29	0	0.587
Hypertension (yes vs. no)	2	264	fixed	0.61 (0.26–0.95)	0.16	0	0.693
Concomitant Cis (yes vs. no)	2	4322	fixed	0.88 (0.77–0.98)	0.01	0	0.913
Tumor grade (high vs. low)	7	5256	fixed	1.06 (0.82–1.30)	1.93	0	0.926
Tumor necrosis (yes vs. no)	4	846	fixed	1.21 (0.70–1.72)	1.20	0	0.753
LVI (yes vs. no)	4	4862	Random	1.00 (0.47–1.53)	9.31	67.8	0.025
Tumor stage (≥T2 vs. <T2)	6	5096	fixed	1.22 (1.07–1.37)	3.26	0	0.661
Multifocality	2	524	fixed	1.33(0.78–1.87)	0.04	0	0.838

### Sensitivity analysis

**Figure 6:**
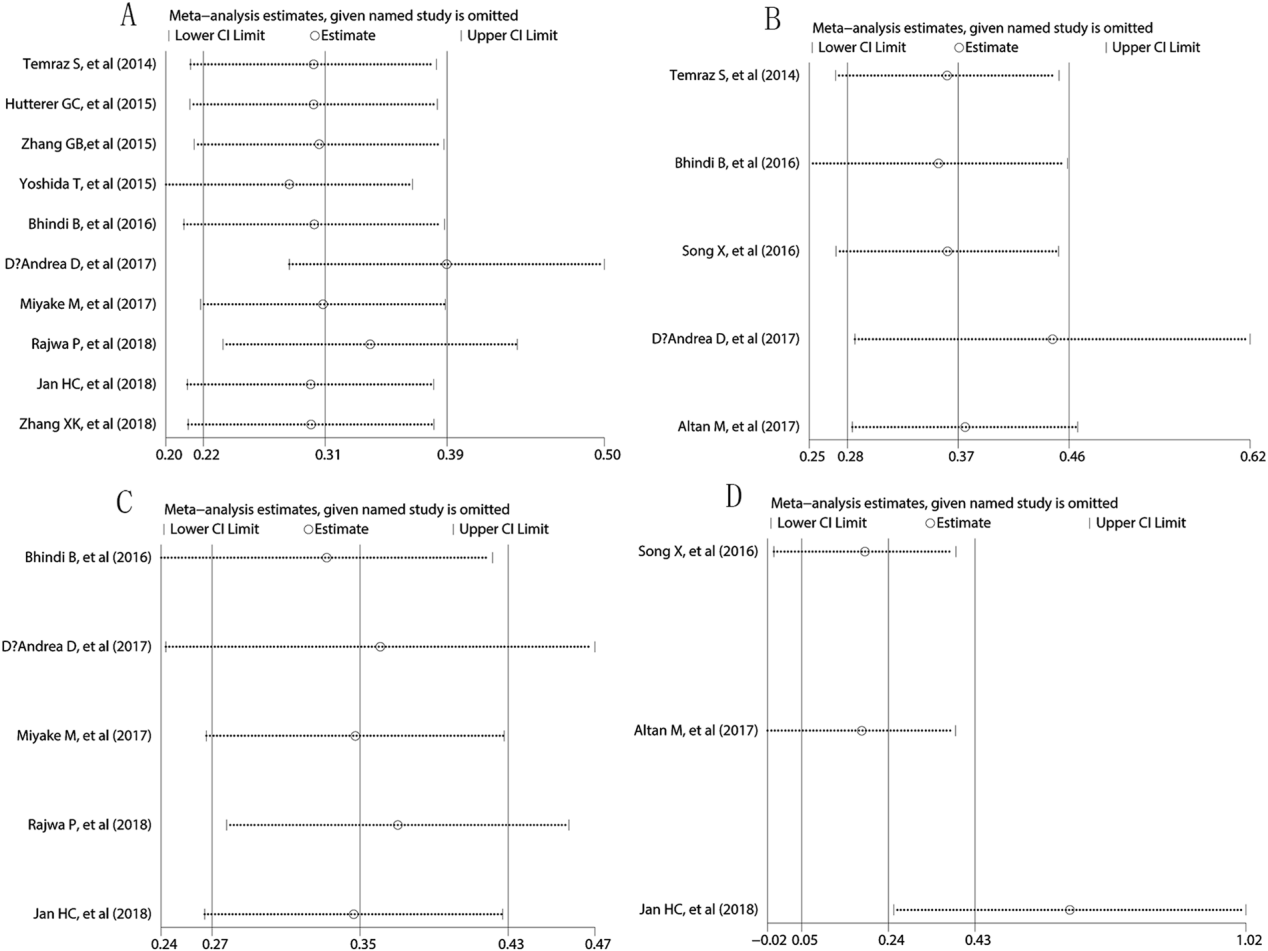
Sensitivity analysis. (**A**) MLR with OS; (**B**) MLR with DFS/RFS; (**C**) MLR with CSS; (**D**) MLR with PFS. Circles: included studies; Dash line: 95% confidence interval.

Sensitivity analysis was performed to evaluate the impact of each single study on the final results. The sensitivity analysis has not found any study contributed significantly to the origin of heterogeneity among the included studies: MLR and OS (Fig. 6A), MLR and DFS/RFS (Fig. 6B), MLR and CSS (Fig. 6C). However, the sensitivity analysis indicated Jan HC *et al*.’s study may be the main origin of heterogeneity regarding the pooled analysis about MLR and PFS. After removal of Jan HC *et al*.’s study, the pooled result also indicated there was no significant association between preoperative MLR and PFS (HR = 2.40, 95%CI = 0.74–4.05, I^2^ = 0.0%).

## Discussion

Urothelial carcinoma was the most common malignant tumors in urinary system, which derived from the lining surface epithelium termed as “urothelium” with the same embryologic origin^1^. UCB and UUTUC were the two main types in urothelial carcinoma with respect to anatomical location, however, the two types behave identically, and much of clinical decision-making could be similar between UCB and UUTUC^29,30^. In order to improve the management of urothelial carcinoma, it is pivotal to identify novel, economic and feasible predictors for individual therapy. Emerging evidences have shown immune cells were involved with cancer initiation, progression and invasion in multiple tumors^31,32^. The hematological inflammatory cells and factors have been widely investigated and identified as important prognostic indicators in urothelial carcinoma^10,33^. More recently, preoperative MLR, as an inflammation related marker, was reported as a potential predictor for urothelial maligancies in several studies. However, there was no related meta-analysis, which can increase the statistical power by methodologically combining the reported results from varying studies. To the best of our knowledge, this is the first pooled analysis to assess the preoperative MLR as a kind of predictor in urothelial maligancies.

According to the pooled analysis of comparative studies from meta-analysis, our results indicated elevated preoperative MLR was significantly associated with reduced OS, DFS/RFS and CSS in urothelial carcinoma patients. However, no significant association was found between preoperative MRL and PFS in urothelial carcinoma patients. Furthermore, subgroup analysis was performed according to the different sites of urothelial carcinoma(UCB, UUTUC), and the results also suggested elevated preoperative MLR predicted worse OS, DFS/RFS and CSS in UCB or UUTUC, respectively. The relationship between elevated preoperative MLR and selected clincopathological features was also assessed in this pooled analysis, and the results demonstrated elevated preoperative MLR was an independent risk for high tumor stage (≥T2 vs. <T2) in urothelial maligancies (OR = 1.201, 95%CI = 1.047–1.356, I^2^ = 0). Collectively, the pooled data from this meta-analysis showed that MLR may serve as a prognostic indicator in urothelial carcinoma patients.

The underlying mechanisms involved with the prognostic role of hematological MLR in urothelial carcinoma remain to be addressed. Mounting studies have indicated tumor infiltrating lymphocytes (TILs) were associated with prognosis of urothelial carcinoma^34,35^, and the prognostic role may vary according to the subpopulations of TILs^35^. Low lymphocyte counts can lead to the deficiency of host immune response, which may cause the progression and metastasis of cancer cells and result in poor survival in malignancies^36,37^. Circulating monocytes can be recruited into tumor microenviroment and further polarize into tumor associated macrophages (TAMs). Elevated tumor infiltrating TAMs were always involved with worse survival in various tumors^38,39^. Taken together, we postulated that elevated preoperative MLR could reflect the disorders of immune cells in tumor miroenvironment, which generated poor survival in urothelial carcinoma patients.

This pooled analysis also had some disadvantages. First, considerable heterogeneity was found in our pooled analysis. In order to detect and decrease the potential heterogeneity, we have performed sensitivity analysis and subgroup analyses. Second, the number of included studies was limited, and the pooled results should be cautiously interpreted. Moreover, 8 included studies had reported HRs and 95%CIs derived from multivariate analysis, while HRs and 95%CIs in 4 studies were extracted from univariate analysia, which may overestimate the prognostic role of MLR. In addition, all the included studies were retrospective design. In future, large-scale prospective studies are still needed to validate our results in this pooled analysis.

Collectively, our priamry results derived from pooled analysis suggested elevated preoperative MLR was significantly correlated with poor survival, which may contribute to the risk stratifications before surgery and clinical decision-making for individual therapeutic strategies in urothelial carcinoma patients.

## Materials and Methods

This pooled analysis was performed according to the Preferred Reporting Items for Systematic Reviews and Meta-Analyses statement (PRISMA)^40^, and the PRISMA checklist was detailed in Supplementary Table S1.

### Search Strategies

There databases (Pubmed, Embase and Web of science) were systematically searched for eligible studies involved with preoperative MLR in urotheilal malignancies, up to November 30^th^, 2018. The selected keywords were confined in the followings: (urothelial cancer OR bladder cancer OR bladder malignancies OR transitional cancer OR ureteral cancer OR renal cancer) AND (MLR OR monocyte to lymphocyte ratio OR monocyte lymphocyte ratio). References listed in identified studies were also went through to expand the scope of search.

### Study selection

#### Inclusion criteria

The studies were included according to the criteria listed in the following: (1) patients were pathologically diagnosed as UCB or UUTUC. (2) MLR was collected before surgery. (3) the studies provided HRs or Kaplan-meier curve for evaluating the role of MLR in prognostic variables. (4) studies reported cut-off values for stratified analysis.

#### Exclusion criteria

The studies were excluded if they met the following criteria: (1) animal studies, editorials, case reports, reviews, comments; (2) duplicates identified by Endnote X7 version; (3) the studies did not focused on the relationship between preoperative MLR and urothelial maligancies or without sufficient data to extract HRs and 95% CIs.

### Data management and quality assessment

Data from the included studies were summarized via a kind of predefined form: Acronym of first author (year of publication), design, geographical region, cases number, diagnosis, therapy, cut-off value, outcome and analysis methods. The related HRs and 95%CIs were extracted to further analyze the association between preoperative MLR and urothelial malignancies. OS, CSS, DFS/RFS and PFS were taken as prognostic outcomes for pooled analysis. The clinicopathological characteristics included gender, diabetes, hypertension, concomitant carcinoma *in situ*, multifocality, tumor grade, tumor necrosis, lymphovascular invasion and tumor stage.

The Newcastle-Ottawa Scale (NOS) system was used to evaluate the quality of included studies^41^. The maximum score is 9, which was involved with the evaluation of subject selection, comparability of groups, and clinical outcome. If the NOS scored more than 7 (included 7) for a study, it was usually taken as a high-quality study. All the processes about data management and quality assessment were reviewed independently by two authors, and any disagreement was settled through discussion with a senior author.

### Statistical analysis

The pooled HRs or ORs were analyzed with Stata version 15.0 (StatCorp, College Station, TX, USA). ORs and 95%CIs regarding the association between preoperative MLR and chinicopathological characteristics were calculated from raw data reported in the included studies. Heterogeneity among the studies was reported via the statistic effects, such as Chi-squared tests, p value and I-square. A fixed-effect model was preferentially used for this pooled analysis, unless there was significant heterogeneity among the studies(I^2^ > 50%). Otherwise, a random-effect model was adopted as an alternative method. An observed HR >1, combined with 1 not included in its 95%CIs, indicated elevated preoperative MLR was associated with poor prognosis in urothelial carcinoma patients. We did not perform Begg’s and Egger’s tests to evaluate the publication bias for that the number of included studies was less than 10 in this pooled analysis^42–44^. However, we conducted sensitivity analysis to validate the pooled results by excluding each study.

## Supplementary information

Supplementary Table S1
